# Investigation of the impact of clonal hematopoiesis on severity and pathophysiology of COVID-19 in rhesus macaques

**DOI:** 10.3389/fvets.2023.1182197

**Published:** 2023-07-06

**Authors:** Tae-Hoon Shin, Yifan Zhou, Byung-Chul Lee, So Gun Hong, Shayne F. Andrew, Barbara J. Flynn, Matthew Gagne, John-Paul M. Todd, Ian N. Moore, Anthony Cook, Mark G. Lewis, Kathryn E. Foulds, Robert A. Seder, Daniel C. Douek, Mario Roederer, Cynthia E. Dunbar

**Affiliations:** ^1^Translational Stem Cell Biology Branch, National Heart, Lung and Blood Institute, National Institutes of Health, Bethesda, MD, United States; ^2^Department of Laboratory Animal Medicine, College of Veterinary Medicine and Veterinary Medical Research Institute, Jeju National University, Jeju, Republic of Korea; ^3^Haematological Cancer Genetics, Wellcome Trust Sanger Institute, Cambridge, United Kingdom; ^4^Wellcome-MRC Cambridge Stem Cell Institute, University of Cambridge, Cambridge, United Kingdom; ^5^Vaccine Research Center, National Institute of Allergy and Infectious Diseases, National Institutes of Health, Bethesda, MD, United States; ^6^Division of Pathology, Yerkes National Primate Research Center, Emory University School of Medicine, Atlanta, GA, United States; ^7^Bioqual, Inc., Rockville, MD, United States

**Keywords:** clonal hematopoiesis, COVID-19, SARS-CoV-2, rhesus macaque, aging, hyperinflammation

## Abstract

Clinical manifestations of COVID-19 vary widely, ranging from asymptomatic to severe respiratory failure with profound inflammation. Although risk factors for severe illness have been identified, definitive determinants remain elusive. Clonal hematopoiesis (CH), the expansion of hematopoietic stem and progenitor cells bearing acquired somatic mutations, is associated with advanced age and hyperinflammation. Given the similar age range and hyperinflammatory phenotype between frequent CH and severe COVID-19, CH could impact the risk of severe COVID-19. Human cohort studies have attempted to prove this relationship, but conclusions are conflicting. Rhesus macaques (RMs) are being utilized to test vaccines and therapeutics for COVID-19. However, RMs, even other species, have not yet been reported to develop late inflammatory COVID-19 disease. Here, RMs with either spontaneous DNMT3A or engineered TET2 CH along with similarly transplanted and conditioned controls were infected with SARS-CoV-2 and monitored until 12 days post-inoculation (dpi). Although no significant differences in clinical symptoms and blood counts were noted, an aged animal with natural DNMT3A CH died on 10 dpi. CH macaques showed evidence of sustained local inflammatory responses compared to controls. Interestingly, viral loads in respiratory tracts were higher at every timepoint in the CH group. Lung sections from euthanasia showed evidence of mild inflammation in all animals, while viral antigen was more frequently detected in the lung tissues of CH macaques even at the time of autopsy. Despite the lack of striking inflammation and serious illness, our findings suggest potential pathophysiological differences in RMs with or without CH upon SARS-CoV-2 infection.

## Introduction

Despite the progress in decreasing morbidity and mortality due to COVID-19 with the advent of antivirals and vaccines, there remain unresolved issues in disease pathophysiology and treatment. While most patients show asymptomatic or self-limited mild illness, a considerable number of patients progress to life-threatening respiratory failure often accompanied by serious complications, including thrombosis, coagulopathy, and cardiovascular disease (CVD). This deterioration often occurs during the second week following infection, with evidence implicating a hyperinflammatory state as associated with poor outcomes (1–3). Extensive studies to date have uncovered several risk factors for severe COVID-19 disease, most strikingly age, as well as gender, ethnicity, underlying genetic variation in immune response pathways, and comorbidities such as diabetes, obesity, and hypertension (4–6). However, even within high-risk groups such as the elderly, outcomes are very heterogeneous and cannot be fully explained by known factors predicting severe illness.

Acquired somatic mutations in hematopoietic stem and progenitor cells (HSPCs) are associated with an *in vivo* competitive advantage and certain human health outcomes including myeloid malignancies. This syndrome is termed clonal hematopoiesis (CH) and greatly increases with advanced age, in a pattern that mirrors the association between age and COVID-19 severity. Of note, loss-of-function (LOF) mutations in certain genes such as the epigenetic regulators DNMT3A and TET2 are the most frequent CH mutations and have been linked to a distinct myeloid cell phenotype associated with hypersecretion of inflammatory cytokines and an increased risk of CVD (7–9). Thus, we and others have hypothesized that the presence of CH could influence the risk of severe COVID-19 disease. Although several human cohort studies have examined this relationship, results to date are conflicting and difficult to interpret due to insufficient sample size, inconsistent sequencing platforms, inclusion of cancer patients with competing risks for CH, and limited availability of information on other potential risk factors (10–14). This potential relationship has not yet been studied in a preclinical model.

Various animal models for COVID-19 have been developed, although no one model closely mimics the course or severity of human disease [reviewed in (15, 16)]. Old world monkeys, including rhesus macaques (RMs), have been established as the most relevant model for SARS-CoV-2 infection and are being widely utilized for preclinical development of therapeutics and vaccines (17, 18). However, preclinical models that recapitulate the pathophysiology of severe or fatal human disease with late hyperinflammation do not yet exist. Macaques experimentally infected with SARS-CoV-2 do not succumb to the infection or develop serious hyperinflammatory clinical manifestations.

We previously identified naturally occurring CH in aged RMs carrying a spectrum of mutations and an incidence similar to human CH (19). In addition, given the lack of availability of naturally aged macaques, we created a CRISPR/Cas genetically engineered CH model in younger adult macaques that reproduces the expansion of clones with TET2 LOF and hyperinflammation (19). In this pilot study, we utilized our macaque models to ask whether disease severity and biologic characteristics following SARS-CoV-2 inoculation was enhanced in animals with either naturally occurring CH expanding post-autologous transplantation or engineered CH mutations in the *DNMT3A* or *TET2* genes introduced via *ex vivo* editing followed by autologous transplantation, in comparison to similarly transplanted and conditioned middle-aged adult animals without CH mutations.

## Materials and methods

### SARS-CoV-2 challenge in rhesus macaques with clonal hematopoiesis

All procedures were approved by the Animal Care and Use Committee of the National Heart, Lung, and Blood Institute (#H-0136) and Vaccine Research Center (#VRC-20-0865). Infection experiments were conducted in animal biosafety level 3 facility at Bioqual, Inc. (Rockville, MD).

Previously, we identified macaques with age-related spontaneous CH by error-corrected ultra-deep sequencing on a panel of 56 common human CH genes. We also created an engineered CH model in younger macaques via CRISPR/Cas genome editing of CD34^+^ HSPCs targeting *DNMT3A*, *TET2*, and *ASXL1* loci, the most frequent mutations observed in human CH, followed by autologous transplantation (19). Both natural and engineered CH animals were confirmed to have characteristic expansion of CH clones over time, and associated phenotypes such as hyperinflammation (19).

One aged macaque with natural DNMT3A CH and two with engineered TET2 CH, along with three control animals similarly transplanted and conditioned, were inoculated with SARS-CoV-2 (USA-W1/2020 strain). Animals were challenged with 8 × 10^5^ plaque-forming units (PFU) total of SARS-CoV-2 via intranasal and intratracheal routes as described previously (17) and monitored until 12 days post-inoculation (dpi).

### Clinical assessment following infectious challenge

**Figure 1:**
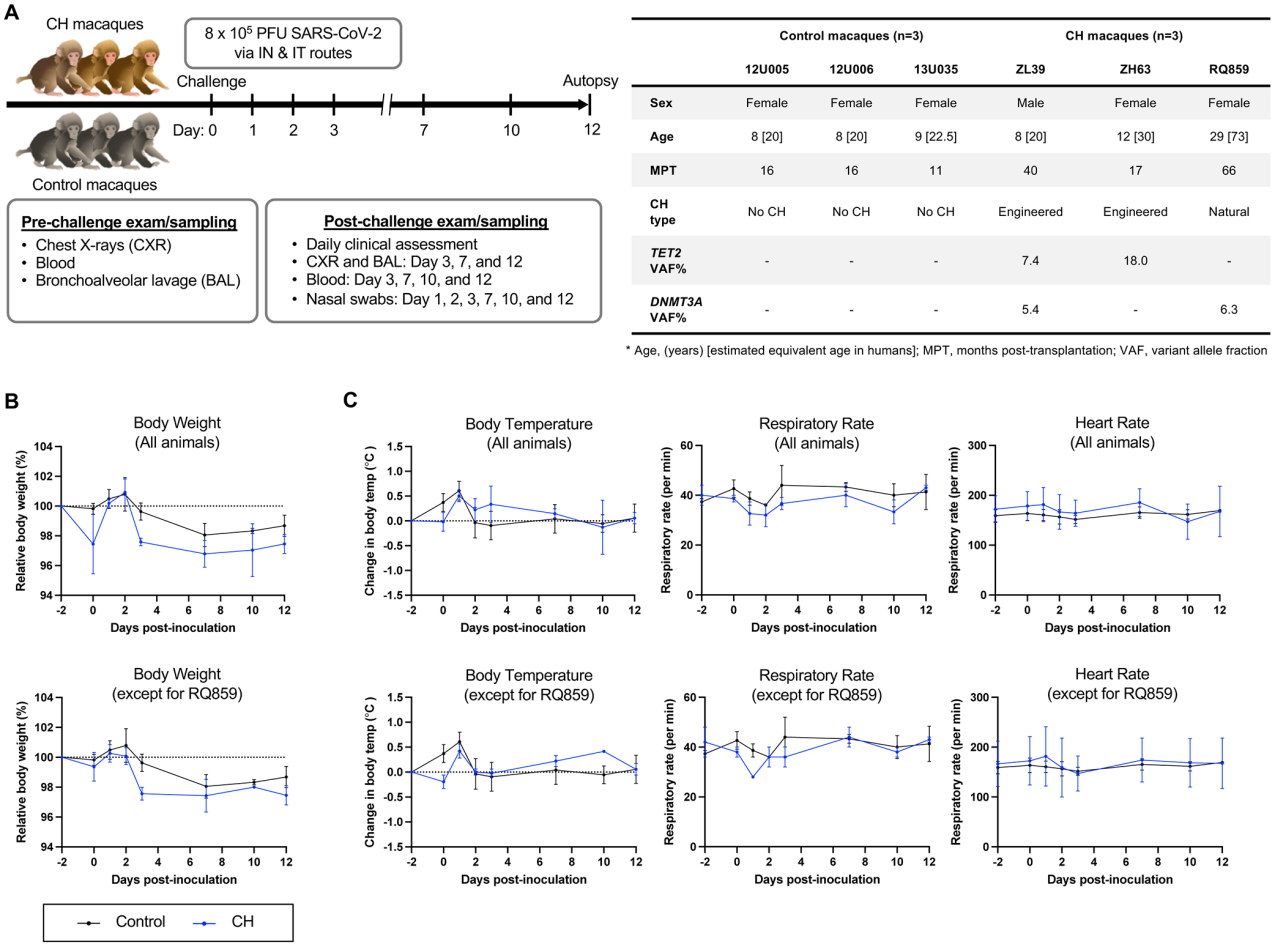
SARS-CoV-2 challenge in a RM model of clonal hematopoiesis. **(A)** Schematic workflow of the experimental procedures, including SARS-CoV-2 inoculation, clinical and radiographic examinations, and specimen collection. Additional information regarding macaques enrolled in this study at the time of the SARS-CoV-2 inoculation is summarized in the table. The number in brackets in the age row gives the estimated equivalent age in humans. In control animals, the absence of CH somatic mutations was verified by error-corrected ultra-deep exome sequencing of a 56 gene panel associated with myeloid neoplasms (19). All animals received myeloablative total body irradiation conditioning prior to autologous transplantation. All animals had normal hematopoiesis as assessed by blood counts and marrow morphology at study entry, however, animal RQ859 was unexpectedly found to be severely anemic on baseline sampling taken at the time of inoculation (Supplementary Figure S4). IN, intranasal; IT, intratracheal. **(B)** Relative body weight changes following inoculation with (top) or without RQ859 (bottom). **(C)** Body temperature changes and changes in respiratory and heart rates between CH and the control group were plotted over time after the SARS-CoV-2 challenge.

Strategies for exams and specimen collections are detailed in Figure 1A. Briefly, cage-side clinical assessment was performed daily with a detailed scoring sheet following the SARS-CoV-2 challenge (Supplementary Table S1). Animals were anesthetized for chest X-ray, complete blood count, blood and bronchoalveolar lavage fluid (BALF) draws on 2 days pre- and 3, 7, 10, and 12 days post-inoculation (dpi) and for nasal swabs on days 1, 2, 3, 7, 10, and 12. During anesthesia, vital indices such as body weight, temperature, and respiratory and heart rates were monitored.

### Histopathology

Lung, spleen, and lymph node samples were collected from SARS-CoV-2-infected macaques during autopsy performed on day 10 (RQ859) and 12. The tissues were fixed in buffered formalin, processed with tissue processor, embedded in paraffin, and sectioned serially at 5 μm. Subsequently, the sections were stained with hematoxylin and eosin (H&E). Immunohistochemical (IHC) analysis was performed with formalin-fixed paraffin-embedded lung sections obtained from at least three different lobes per animal. As described previously (17), the slides were stained with a rabbit polyclonal SARS-CoV-2 (GeneTex, Irvine, CA) using the BOND-RX Multiplex IHC Stainer (Leica Biosystems, Wetzlar, Germany), and DAB chromogen was detected using the Bond Polymer Refine Detection Kit (Leica Biosystems). Microscopic evaluation of each staining was conducted by a board-certified veterinary pathologist. Inflammation and viral detection scores were calculated according to the following scales: (1) 0 = absent, 1 = minimal to mild, 2 = mild to moderate, 3 = moderate to severe, 4 = severe pulmonary inflammation, (2) 0 = non-detected, 1 = rare to occasional, 2 = occasional to multiple, 3 = multiple to numerous, 4 = numerous SARS-CoV-2 antigens.

### Viral loads

To quantify SARS-CoV-2 replicating in the respiratory tract, respiratory tract, polymerase chain reaction (PCR) was performed to detect subgenomic RNAs (sgRNAs) of both envelope (E) and nucleocapsid (N) as described previously (17, 20). Briefly, total RNA was extracted from nasal swabs and BALF using RNAzol BD column kit (Molecular Research Center, Cincinnati, OH) according to the manufacturer’s instruction. PCR reactions were conducted using TaqMan Fast Virus 1-Step Master Mix (Applied Biosystems, Waltham, MA), gene-specific probes, forward primer in the 5′ leader region, and reverse primers. Amplification of sgRNAs was calculated using the QuantStudio 6 Pro Real-Time PCR System (Applied Biosystems), with a lower detection limit of 50 copies per reaction.

### Cytokine quantification

Serum was separated from cellular components of each blood sample by centrifugation at 4°C. BALs were concentrated 10X prior to cytokine quantification. Concentrations of major cytokines and chemokines in serum and BAL were measured using MILLIPLEX MAP Nonhuman Primate Cytokine Magnetic Beads Panel (Millipore Sigma, Burlington, MA) according to the manufacturer’s instructions. Luminex data were analyzed using MAGPIX with Bio-Plex Manager^™^ MP software (Bio-Rad, Hercules, CA).

### Clonal tracking

Through density gradient centrifugation, mononuclear cells and granulocytes were isolated from peripheral blood (PB) and bone marrow (BM). Cellular components of BAL were separated from fluid after centrifugation. Genomic DNAs extracted from the granulocytes and BAL cells were amplified using gene-specific primers for mutated regions at DNMT3A (RQ859) and TET2 (ZL39 and ZH63) and ligated with unique index sequences, followed by sequencing on Illumina Miseq. The sequencing reads with mean depth of more than 300,000 were analyzed using CRISPResso (http://crispresso.rocks) and custom R pipelines as documented previously (19). The remaining portion of the BAL cells were used for immunotyping of the population using flow cytometry.

### Statistical analysis

All statistical analyses were performed using GraphPad Prism 9 (GraphPad Software, San Diego, CA). All graphs with error bars are presented as the mean ± S.E.M. Unpaired student’s *t*-test was applied for pairwise comparisons between two groups, and One-way ANOVAs followed by the Tukey’s *post-hoc* test for comparisons of multiple groups.

## Results

### CH did not affect disease severity following SARS-CoV-2 infection in rhesus macaques

Previously described RMs with spontaneous DNMT3A CH expanding post-autologous transplantation (RQ859) (19, 21) or CRISPR-engineered TET2 CH (ZL39 and ZH63) (19), along with control non-CH animals receiving autologous transplantation following the same total body irradiation conditioning protocol (*n* = 3) were inoculated with SARS-CoV-2 and monitored for 12 days (Figure 1A). All animals manifested only mild signs of clinical illness with scores ranging from 3 to 4 in all animals, without differences between the two groups, and no evidence of significant pulmonary inflammation on repeated chest radiographs (Supplementary Figure S1). However, the natural aged CH animal RQ859 died suddenly on 10 dpi of unclear causes. RMs with CH showed a slower tendency in body weight recovery overall compared to controls, even when excluding animal RQ859 which experienced extreme weight loss and died suddenly (Figure 1B). No remarkable differences were observed in body temperature and heart rates between the groups, regardless of whether RQ859 was included or not (Figure 1C and Supplementary Figure S2).

### Macaques with CH showed a tendency of local hyperinflammation in the lungs

**Figure 2:**
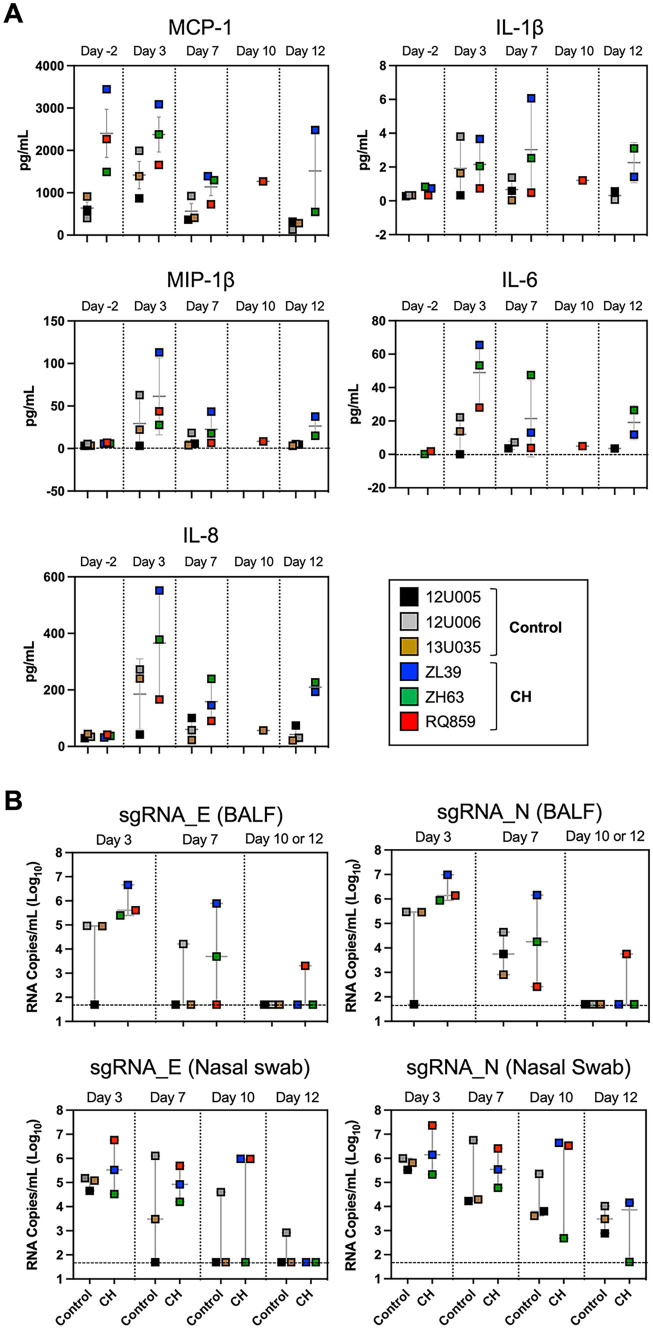
Post-challenge inflammatory response and viral load in macaques with CH. **(A)** Post-challenge BALF cytokine/chemokines levels in CH macaques vs. controls. Box plots depicting the results of immunoassays for rhesus macaque MCP-1, IL-1β, MIP-1β, IL-6, and IL-8. **(B)** Assessment of viral replication in BALF and nasal swab specimens by SARS-CoV-2 envelope (E) and nucleocapsid (N) sgRNA quantification. Each colored symbol indicates individual macaques and the dashed horizontal lines show the limit of detection for the assay.

To evaluate post-challenge inflammatory responses in the presence or absence of CH, sera and BALF cytokine/chemokine concentrations were measured serially. In BALF, mean concentrations of MCP-1, IL-6, IL-8, and MIP-1β were consistently higher in CH macaques compared to controls (Figure 2A), whereas no constant patterns in serum cytokine/chemokine levels were observed between the two groups, except for somewhat higher IL-6 concentration in CH animals until 12 dpi (Supplementary Figure S3), although small numbers of animals precluded statistical significance.

### SARS-CoV-2 infection was not directly associated with change in CH mutation frequency

Given that IL-6 is hypothesized to be a major factor contributing to the vicious cycle between hyperinflammation and clonal expansion in TET2 and DNMT3A CH (22–24), we asked whether the elevated levels of lung IL-6 in CH macaques resulted from or contributed to local lung expansion or persistence of CH mutant cells following SARS-CoV-2 infection. Targeted deep sequencing measured variant allele frequencies (VAFs) for specific CH mutations in both BALF cells and granulocytes isolated from peripheral blood and bone marrow. No significant changes were observed in peripheral blood or marrow granulocytes, whereas mutation VAFs in the cellular component of BALF shifted in both directions following infection (Supplementary Figure S4), possibly due to changes in neutrophil to lymphocyte ratio with infection, since CH mutation VAFs are usually lower in lymphoid cells (19).

### Presence of CH augmented the persistence and replication of SARS-CoV-2 in macaques

**Figure 3:**
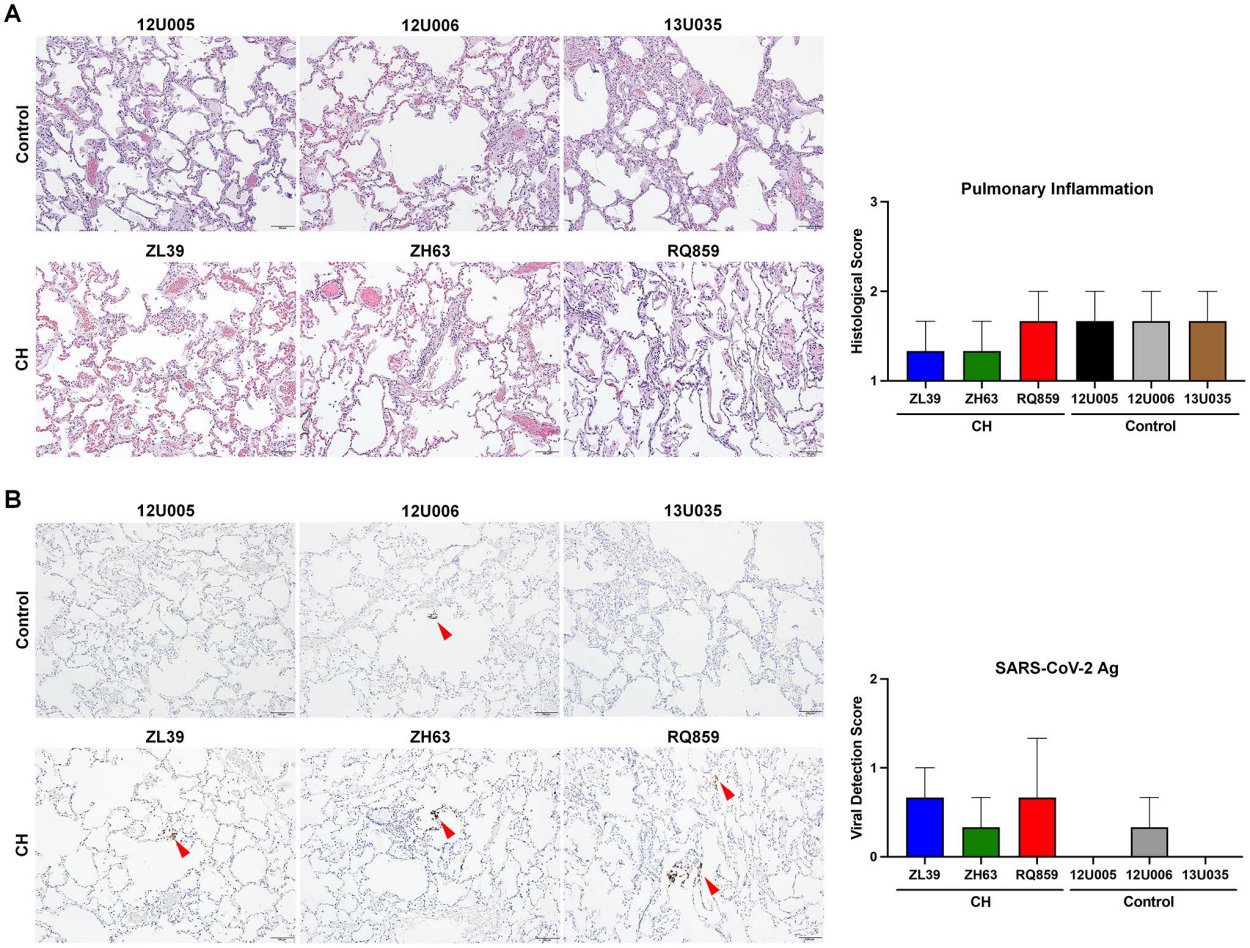
Histopathologic and immunohistochemical analyses of the lung tissue **(A,B)**. To assess the degree of pulmonary inflammation and virus survival, the lung tissues of RMs with or without CH isolated at 12 dpi were stained with H&E **(A)** and an antibody specific for SARS-CoV-2 antigen (Ag) **(B)**, respectively. Representative microscopic images are shown on the left side of each panel (scale bar = 100 μm), and histopathologic and viral detection scores were calculated based on the defined criteria and graphed on the right. Red arrowheads in **(B)** indicate the detected viral antigens in the lung tissues.

To assess viral replication in the upper and lower airways, we measured levels of sgRNAs encoding SARS-CoV-2 envelope and nucleocapsid in nasal swabs and BALFs collected at 3, 7, 10, and/or 12 dpi. Interestingly, the median copy numbers of both sgRNAs were higher at every timepoint in the CH group compared with controls, with more substantial differences in BALF (Figure 2B). Histopathologic analysis on the lung sections from euthanasia at 10 or 12 dpi found evidence for mild inflammation in all animals (Figure 3A). Confirming the viral load measurements, viral antigen was immunohistochemically detected in the lung tissues of all three CH animals but only one of three controls (Figure 3B). No remarkable differences were found in other tissues such as lymph nodes and spleen, and we did not find any atherosclerotic changes in the hearts of the CH animals.

## Discussion

Our results showed potential differences in the pathophysiology of SARS-CoV-2 infection in RMs, however, the differences were not statistically significant overall, which is not surprising considering the small sample size. The insufficient number of animals is undoubtedly a limitation in reaching firm conclusions, but considering the limitations on performing complex studies during the COVID-19 pandemic, our results at least provide the core evidence to prove our hypothesis, in parallel with the large human cohorts (14).

The naturally aged CH animal died suddenly in the middle of the experimental period. Although we could not find definitive evidence of this premature death from clinical and histopathological analyses, the event may have been owing to the animal’s very old age (29 years) and unexpectedly severe baseline anemia of unclear etiology present at the time of viral challenge and unfortunately not detected on earlier sampling (Supplementary Figure S5). A contribution from SARS-CoV-2 infection to the demise of this animal cannot be ruled out. Given the possibility of another cause for mortality, we presented the data on vital indices such as body weight and temperature both excluding this animal and including the entire data set. We showed all the results for this animal despite the difference in age and type of CH because we expected this animal itself to provide scientific clues for the correlation between CH and COVID-19 disease severity.

A recent study by Speranza et al. demonstrated more sustained local inflammatory responses in older RMs than in younger RMs, despite similar disease outcomes (25). Consistent with this study, we observed a trend toward sustained high concentrations of key inflammatory cytokines and chemokines in the local and systemic environments following the SARS-CoV-2 challenge in macaques with CH, regardless of the difference in the degree of clinical symptoms. These findings suggest the possibility of CH-specific divergence of immune responses against SARS-CoV-2 even at similar ages. The prior study did not screen for the presence of CH in the older macaques, and our study did not include aged macaques without CH.

Despite the absence of remarkable signs of inflammation and illness, macaques with CH consistently showed higher copy numbers of SARS-CoV-2 sgRNAs at every timepoint in both nasal swabs and BALF and longer persistence, which suggests that CH might impact the extent of viral replication in both the upper and lower respiratory tracts. Moreover, viral antigens were persistently maintained up to 10- or 12-days post-inoculation in all three monkeys with CH, contrary to control animals with infrequent detection. Although the relationship between viral load or persistency and the severity of COVID-19 disease has not yet been established with certainty (26–28), our findings, together with a recent study showing a higher level and longer period of SARS-CoV-2 RNA detection in elderly macaques (29), are relevant to considering the relationship between aging, CH, and COVID-19.

In summary, our nonhuman primate model of CH demonstrates its possible utility as a preclinical tool for investigating the relationship between CH and other diseases such as COVID-19. Consistent with our recently completed study in large human cohorts (14), we found no evidence for a direct correlation between CH and severity of clinical COVID-19 in RMs, although data from this small cohort does provide interesting preliminary evidence for potential pathophysiological differences in macaques with or without CH upon SARS-CoV-2 infection, particularly regarding the level of inflammatory cytokines/chemokines in the lung, and the degree and persistence of virus in the upper and lower respiratory tracts. The creation of CH in macaques is straightforward, and this model may be useful for hypothesis testing in the future.

## Data availability statement

The original contributions presented in the study are included in the article/Supplementary material, further inquiries can be directed to the corresponding author. The sequencing data presented in this study are deposited in the NCBI SRA repository, accession number PRJNA944168 (BioProject) and SAMN33739481 to 33739529 (BioSample); https://www.ncbi.nlm.nih.gov/sra/PRJNA944168.

## Ethics statement

The animal study was reviewed and approved by Animal Care and Used Committee of the National Heart, Lung, and Blood Institute, Vaccine Research Center, and Bioqual, Inc.

## Author contributions

T-HS and CD conceptualized the study and wrote the manuscript with input from all coauthors. CD, KF, RS, DD, and MR designed and supervised the study. T-HS, YZ, B-CL, SA, BF, MG, IM, and AC performed experiments and analyzed data. SH, J-PT, and ML organized and conducted the animal care, transfer, and infection. All authors contributed to the article and approved the submitted version.

## Funding

This work was supported by grant 2022R1F1A1075100 from the National Research Foundation of Korea (NRF) funded by the Korea government (MSIT), the Division of Intramural Research of the National Heart, Lung, and Blood Institute, and the Vaccine Research Center of the National Institute of Allergy and Infectious Diseases, both at the National Institutes of Health.

## Conflict of interest

The authors declare that the research was conducted in the absence of any commercial or financial relationships that could be construed as a potential conflict of interest.

## Publisher’s note

All claims expressed in this article are solely those of the authors and do not necessarily represent those of their affiliated organizations, or those of the publisher, the editors and the reviewers. Any product that may be evaluated in this article, or claim that may be made by its manufacturer, is not guaranteed or endorsed by the publisher.
