# Supplementary material for: Investigation of the impact of clonal hematopoiesis on severity and pathophysiology of COVID-19 in rhesus macaques
Source: Front Vet Sci. 2023 Jul 6;10:1182197. doi: 10.3389/fvets.2023.1182197 (PMC10358770; doi:10.3389/fvets.2023.1182197)
Supplement: Supplementary file 1 [file Data_Sheet_1.PDF]

Supplemental Material

Investigation of the Impact of Clonal Hematopoiesis on Severity and Pathophysiology of COVID-19 in Rhesus Macaques

Tae-Hoon Shin[1,2,*], Yifan Zhou[1,3,4], Byung-Chul Lee[1], So Gun Hong[1], Shayne F. Andrew[5], Barbara J. Flynn[5], Matthew Gagne[5], John-Paul M. Todd[5], Ian N. Moore[6], Anthony Cook[7], Mark G. Lewis[7], Kathryn E. Foulds[5], Robert A. Seder[5], Daniel C. Douek[5], Mario Roederer[5], Cynthia E. Dunbar[1]

*** Correspondence:** Tae-Hoon Shin: shinlab@jejunu.ac.kr

1 Supplemental Table

**Responsiveness**	
0	Normal – bright, alert, responsive
1	Mildly affected – slightly depressed, act disinterested with personnel in room, lies down in cage but gets up when approached
2	Moderately affected/obtunded – non-responsive, very disinterested in personnel, hunched or lying down, will get up when stimulated
3	Severely affected/comatose – lying down completely unresponsive to stimuli
**Discharges**	
0	Normal
1	Mild nasal/ocular
3	Severe nasal/ocular
**Respiratory rate**	
0	Normal
1	Mild tachypnea
3	Severe tachypnea
**Respiratory efforts**	
0	Normal – no apparent changes in breathing
1	Mild – slightly increased effort breathing
3	Severe – open mouth breathing, abdominal breathing
**Cough**	
0	None
1	Mild
3	Severe

Supplemental Table 1. Criteria for cage-side clinical scoring

Cage-side clinical assessment was performed according to these criteria. The scores of the five categories for each animal were summed and recorded daily.

2 Supplemental Figures

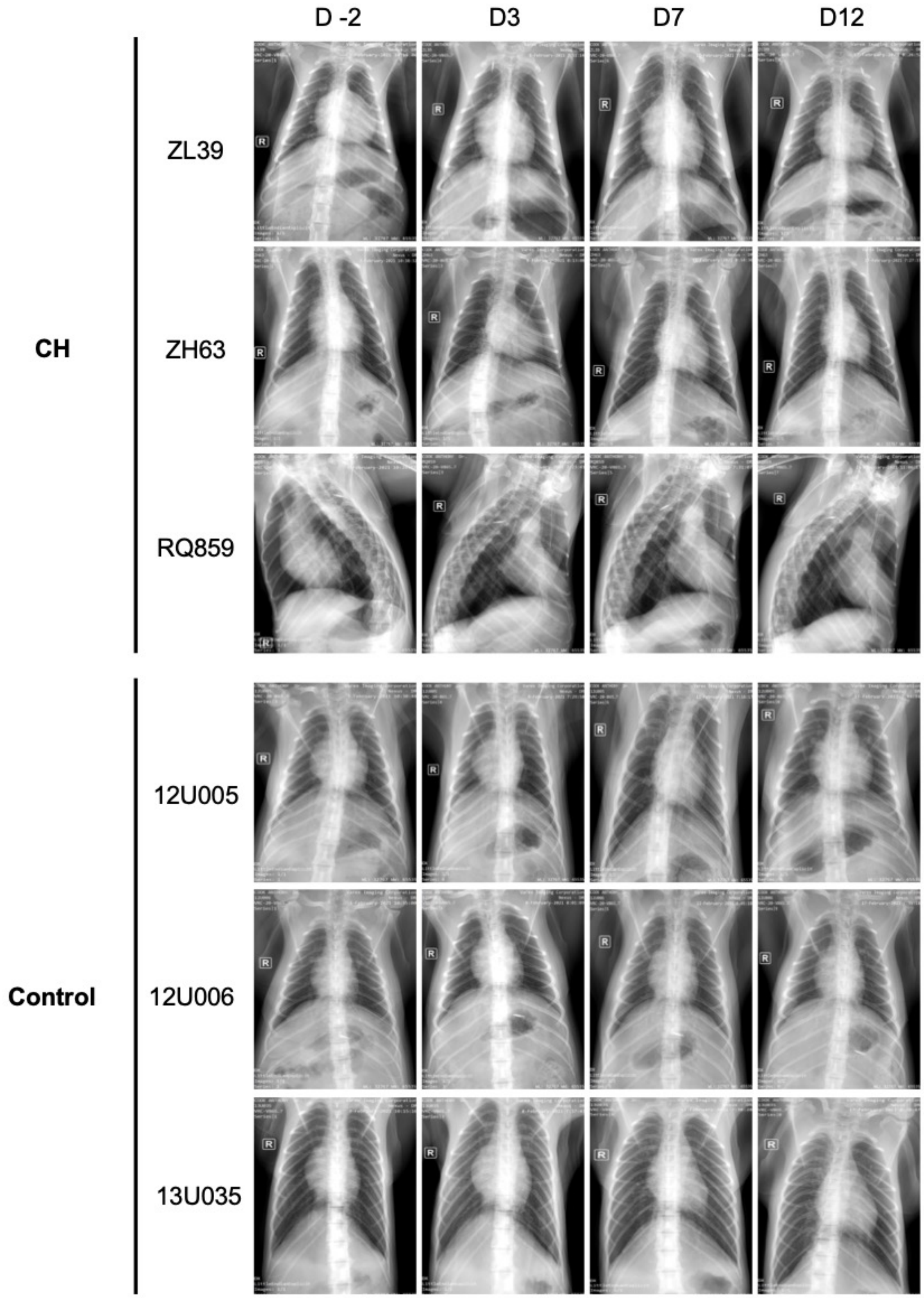

Supplemental Figure 2. Post-challenge chest radiographs in macaques with or without CH.

Chest radiographs were taken repeatedly to assess pulmonary infiltrates upon SARS-CoV-2 inoculation. For RQ859, the image taken on 10 dpi replaces the 12 dpi due to the sudden death before autopsy.

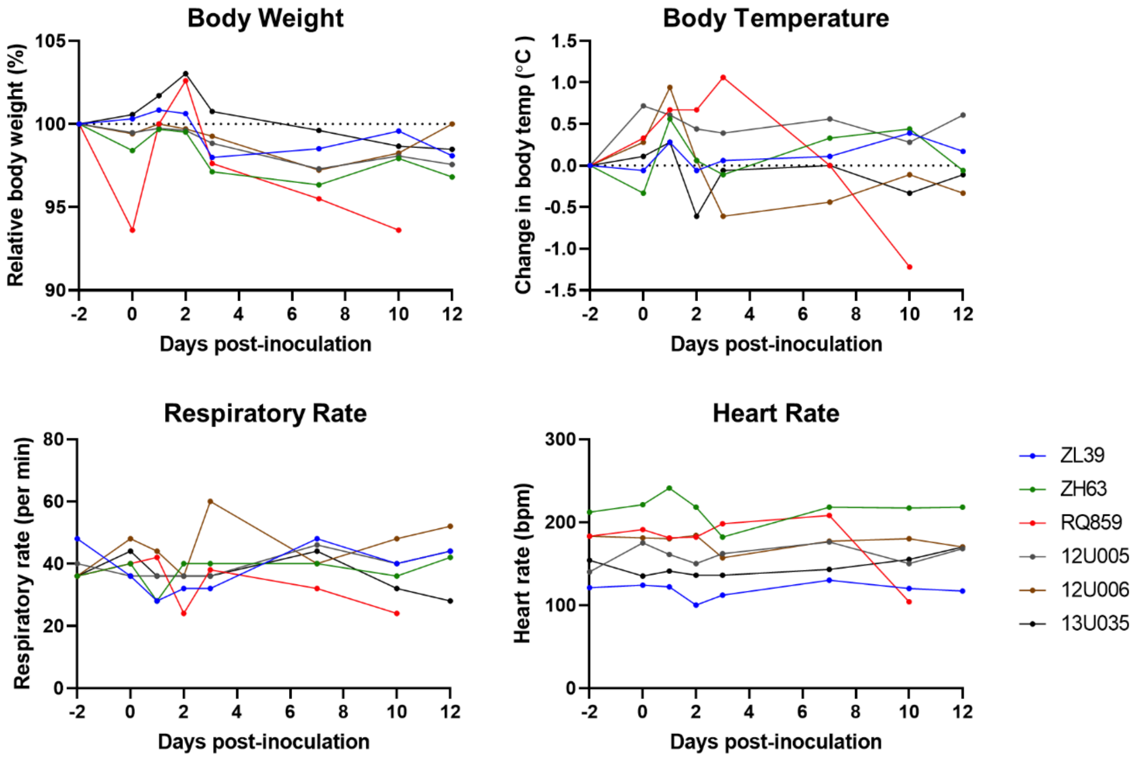

Supplemental Figure 2. Changes in clinical parameters in individual animals over time following the SARS-CoV-2 challenge

Relative changes in body weight and temperature and the absolute number of respiratory and heart rates in individual animals upon SARS-CoV-2 infection were plotted here. All of these data served as the basis for the group comparison plots presented in Figure 1B and 1C.

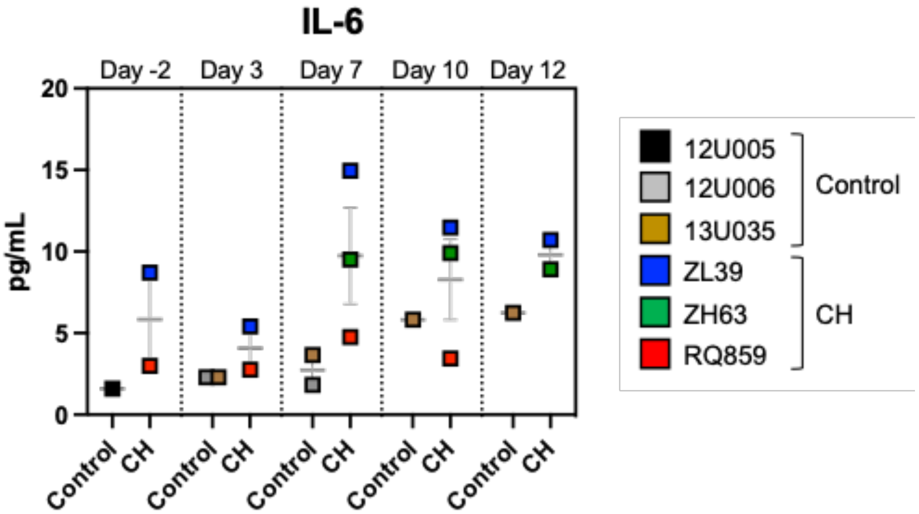

Supplemental Figure 3. Serum IL-6 levels following SARS-CoV-2 infection.

Blood serum samples collected on baseline (-2) and 3, 7, 10, and 12 dpi were analyzed for a panel of 23 chemokines and cytokines using MILLIPLEX® MAP. Among them, the concentration change of macaque IL-6 was depicted here. Data are presented as the mean ± S.E.M. Each colored symbol indicates individual animals, and the dashed horizontal line shows the detection limit of the assay.

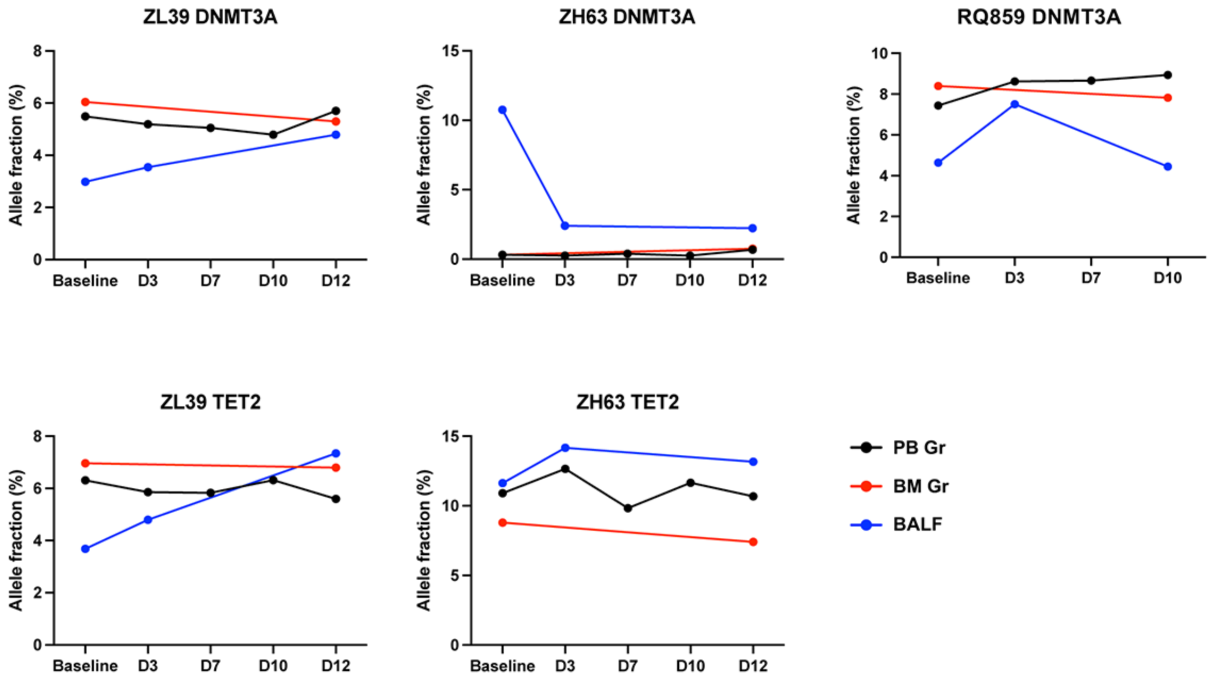

Supplemental Figure 4. Tracking of CH mutations following infection in blood and lung of CH macaques.

Pre- and post-inoculation changes in VAFs of DNMT3A mutations (engineered mutations of ZL39 and ZH63 and spontaneous mutation of RQ859, respectively) and TET2 mutations (ZL39 and ZH63) were analyzed in granulocytes (Gr) from peripheral blood (PB) and bone marrow (BM) and cellular components of BALF using targeted deep sequencing.

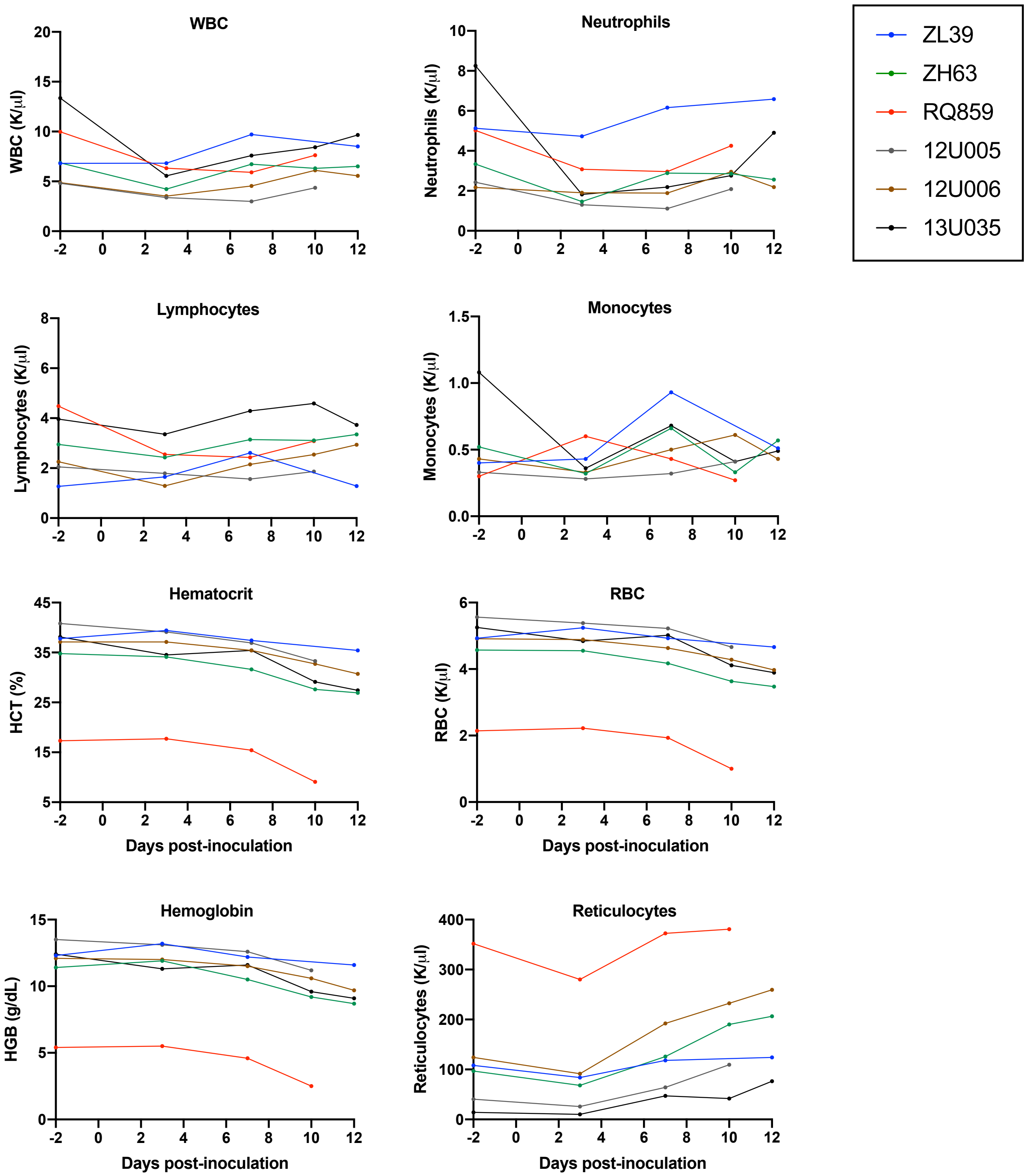

Supplemental Figure 5. Complete blood counts of macaques with or without CH after the SARS-CoV-2 inoculation.

Changes in the primary indices of blood counts before and after challenge were indicated for each animal. RQ859 was unexpectedly severely anemic at the time of the challenge.
